# FANTASTIC lifestyle questionnaire from 1983 until 2022: A review

**DOI:** 10.34172/hpp.2023.11

**Published:** 2023-07-10

**Authors:** Patrícia Batista, João Neves-Amado, Anabela Pereira, João Amado

**Affiliations:** ^1^Universidade Católica Portuguesa, Research Centre for Human Development (CEDH), Human Neurobehavioral Laboratory (HNL), Porto, Portuga; ^2^Universidade Católica Portuguesa, Centre for Interdisciplinary Research in Health (CIIS), Institute of Health Sciences (ICS), Porto, Portugal; ^3^University of Évora, Center for Research in Education and Psychology, Évora, Portugal; ^4^Universidade Católica Portuguesa, Centre for Interdisciplinary Research in Health (CIIS), Porto, Portugal

**Keywords:** Healthy lifestyle, Health behavior, Quality of life, FANTASTIC questionnaire

## Abstract

**Background:**

Studying lifestyles has always been important; quantifying them has become more complex. However, a questionnaire produced in 1983 has shown that its simple form of evaluation can be an added value in understanding lifestyles. Our aim is a systematic review of the scientific literature about the use of the FANTASTIC Lifestyle questionnaire (FLQ).

**Methods:**

The reflective systematic literature review on PubMed, Medline, Science Direct, and SCIELO databases with the descriptors "FANTASTIC Lifestyle questionnaire" OR "FANTASTICO questionnaire" OR "FANTASTIC questionnaire" OR "FANTASTIC survey" OR "FANTASTIC checklist". PRISMA criteria reporting of systematic reviews and meta-analyses were applied. The inclusion criteria were the use of FLQ instrument to measure lifestyles, presenting quantitative or qualitative results, and psychometric studies. It excluded other lifestyle instruments, incomplete articles, and non-English, Brazilian, Spanish, and Portuguese language articles.

**Results:**

Findings reveal 41 scientific articles included in the study. It analyzed the results and most studies use the instrument to assess all dimensions. However, some studies reported assessing specific dimensions such as nutrition, sleep, stress, tobacco, alcohol, and drugs. The questionnaire has been applied to a wide range of ages and literacy levels.

**Conclusion:**

This literature review allowed us to conclude that this questionnaire is still in use today and is applied in several contexts and populations. It is also possible to verify the relevance of its use and to design intervention strategies and programs for a healthy society. It is essential to draw attention to this issue and promote health literacy (HL) on this topic.

## Introduction

 Community and individual healthy lifestyles and behaviors has always been a concern, a problem that should be invested in to improve the quality of people’s lives and avoid diseases and health management problems. Lifestyles and health behaviors are crucial factors that determine the health of a society.^1^ There is large evidence that some behaviors and lifestyles have the potential of determining direct and/or indirect impact on health, on the physical, mental and social wellbeing of each person. For example, a healthy diet, regular physical activity, moderate alcohol consumption, and drug abstinence are considered healthy lifestyles that can predispose an individual to better health conditions. On the other hand, drug consumption (illegal drugs or tobacco and alcohol), unsuitable diet, sedentary lifestyle, and early and/or promiscuous sexual activity, are behaviors that can determine risks for an individual’s health and life.^2^ These unhealthy lifestyles are seen as the main risk factors for chronic diseases and premature deaths and determine an important part of the deaths and illnesses that occur in the European region.^2^

 Thus, diagnosing lifestyles makes perfect sense, in order to be able to intervene afterward. Designing promotion and intervention plans and increasing the health literacy (HL) of the entire population. One of the most important strategies to improve a healthy lifestyle is to increase knowledge and promote HL among people. The World Health Organization (WHO) refers to HL as a key element of health and recommends that countries create a multi-stakeholder “Council on Health Literacy”.^3^

 Therefore, in 1983 Wilson and his team developed the FANTASTIC Lifestyle questionnaire (FLQ) to evaluate the population lifestyles.^4^ This lifestyles evaluation instrument was developed not only to diagnose population lifestyles but for use in a community health education program. Thus, the aim of this study is to provide an overview of the scientific evidence for using this specific instrument “FANTASTIC questionnaire” to assess lifestyles.

###  FANTASTIC Lifestyle questionnaire 

 In 1983, Wilson and collaborators had a marked interest in health promotion and developed FANTASTIC Lifestyle Assessment.^4,5^

 The FANTASTIC questionnaire incorporates physical, emotional, and social lifestyle factors.^4^ The origin of the word “FANTASTIC”, is the acronym of the names of the 9 dimensions in which 28 items are distributed: F = Family and friends (2 items); A = Activity and Associativity (3 items); N = Nutrition (3 items); T = Tobacco (2 items); A = Alcohol and other substances (6 items); S = Sleep and stress (3 items); T = Type of personality (3 items); I = Introspection (3 items); C = Control of health (3 items). More recently, in some countries the FANTASTIC update to FANTASTICO, was increase one more dimension, O = Other conducts (2 items).^6,7^ Each item has three Likert-type response options.^5,6^

 The questionnaire was first used at McMaster for clinical applications and as a survey instrument in planning health promotion services.^5,8^ FANTASTIC lifestyle assessment was a good instrument to help the general practitioner to assess and to promote healthy lifestyles in their patients. Nowadays, it is also valuable for exploring lifestyle and health. The FANTASTIC lifestyle assessment is a fairly reliable tool, and it is a simple method for people to quickly assess lifestyle behaviors.^5^ This instrument has been used in different countries, due to the speed and ease of filling.

## Materials and Methods

 We conducted a study of the reflective systematic literature review. We found several scientific papers published in international journals, using a search of the databases in digital format, Medline, PubMed, Science Direct and SciELO. The descriptors used in the research were: [FANTASTIC Lifestyle questionnaire” OR “FANTASTICO questionnaire” OR “FANTASTIC questionnaire” OR “FANTASTIC survey” OR “FANTASTIC checklist”]. From the 105 articles found, the duplicates and those that did not meet the inclusion criteria were removed and we obtained 41 articles. We found others publication, but not indexed in these databases.

###  Inclusion criteria

 All studies that used the FLQ, presented/analyzed quantitative/qualitative data, and psychometric studies of psychometric validation of scale/instrument were eligible to include in the study. Also, it included articles written in English, Brazilian, Spanish and Portuguese languages.

###  Exclusion criteria

 Studies with the following criteria were excluded: did not used the FLQ and did not present/analyze quantitative/qualitative data.

**Figure 1:**
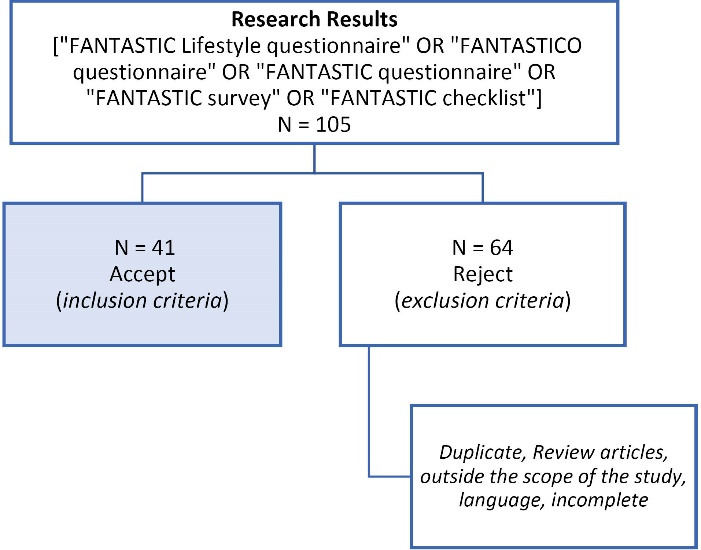
Flowchart - applying the inclusion and exclusion criteria in research

 Preferred Reporting Items for Systematic Reviews and Meta-Analyses (PRISMA) criteria were applied (Figure 1). The information collected was compiled and analyzed in relation to the year of publication, authors, sample, country, type of study/methodology, results and aim. The cataloging and identification of repeated references were made through the computer program EndNote bibliographic referencing.

## Results

 For a better understanding of the systematic literature review, the studies analyzed were compiled in a summary table (Table 1). In this table, they were several items: year of publication/authors, sample, country, methodology, instruments, domains of FANTASTIC, results and aims.

**Table 1:** Summary of information from 41 relevant articles met our inclusion criteria in the study of “FANTASTIC lifestyle questionnaire”

**First author, Year, References**	**Sample**	**Country**	**Methodology**	**Instruments**	**FLQ (dimensions evaluated)**	**Main Results**	**Aim**
Borges, 2022^9^	N = 634 adolescents	Brazil	Cross-sectional study	- FLQ	- Nutrition, - Tobacco, - Sleep	- Anthropometric indicators of body adiposity had greater discriminatory power of HBP screening in males. - For females anthropometric indicators showed area under the curve values (95% CI) below 0.60.	- to propose cutoff points for anthropometric indicators for HBP screening in adolescents and to identify, among these indicators, those more accurately for boys/girls.
González, 2022^10^	N = 407 elderly people	Colombia	Quantitative approach, descriptive type, non-experimental design, cross-sectional	- FLQ	- ALL	- 53% of elderlies reported their lifestyle as excellent and very good. - Age, average household income, and perceived health status are associated with healthy lifestyles in older adults.	- to establish the relationship between socioeconomic factors and healthy lifestyles in older adults
Martins, 2022^11^	N = 61 participants	Brazil	Cross-sectional exploratory study	- FLQ	- ALL	- The model composed by the 3 factors observed in this group of students represented the construct quality of life, evaluated by the FLQ. - This result may provide substrate to actions that aim to improve quality of life and well-being in medical students from this university.	- to identify what structure represents lifestyle in medical students, in a public university, with the application of the FLQ.
Quinones-Laveriano, 2022^12^	N = 217 teachers	Peru	Observational, quantitative, analytical, cross-sectional study	**-** FLQ **- **TSS	- ALL	- 64% of the teachers had a good-excellent lifestyle; 27.2%, regular; and 8.49%, bad-dangerous. - As stress increased, the lifestyle quality worsened in teachers at some schools, during telework in 2020.	- to determine the correlation between stress and lifestyle in teachers at some schools in Lima, Peru, during telework in 2020.
Ruiz-Zaldibar, 2022^13^	N = 488 university students	Spain	Observational, descriptive, and cross-sectional survey study	- FLQ	- ALL	- Students' lifestyles worsened during the lockdown (women were the most affected). - Social/emotional behaviors were deeply affected, while confinement could be a protective factor against previous toxic habits.	- to investigate the perceived changes in lifestyle behaviors among Spanish university students during COVID-19-related confinement.
Vilovic, 2022^14^	N = 483 family physicians	Croatia	Cross-sectional study	- FLQ - BRS - OBI - SWLS	- ALL	- 32.5% of family physicians disclosed positive MHD history, while 68.7% used professional help. - Resilience and healthy lifestyle levels were significantly higher in MHD negative family physicians, while burnout levels were lower; and then were shown as independent predictors of positive MHD history status.	- to assess MHD history, attitudes, and stress-coping strategies in family physicians. An additional goal was to estimate their comprehensive well-being and investigate connections with resilience and a healthy lifestyle.
Bim, 2021^15^	N = 971 adolescents	Brazil	Cross-sectional study	**-** FLQ - BECCQ - IPAC - SBQ	- Nutrition	- 6 out of 10 adolescents presented low levels of HGS. - main predictors of low levels of HGS in boys were age, physical activity, sedentary behavior, and balanced diet, and weight status and height in both genders.	- to identify (HGS) levels and associated modifiable and non-modifiable risk factors in adolescents
Navarro-Cruz, 2021^16^	N = 639 participants	Chile	Cross sectional study	- FLQ	- ALL	- Different dietary behaviors (mainly consumption of industrialized foods) during lockdown, as well as quality of life deterioration were the main factors associated with self-reported weight gain during lockdown.	- to evaluate the association of differences in dietary behaviors and lifestyle with self-reported weight gain during the COVID-19 lockdown in Chile.
Patel, 2021^17^	N = 439 university students	Poland	Cross-sectional study,	- FLQ	- ALL	- The prevalence of FM in medical students seems to be considerably higher than in the general population. - Chronic stress levels, sleep problems, social support, and behavior seem to be the major factors influencing FM severity in this population.	- to assess the prevalence of FM in this population and identify lifestyle parameters influencing FM severity
Saavedra Espinosa, 2021^18^	N = 28 patients	Colombia	Mixed study (qualitative and quantitative)	- FLQ	- ALL	- The participants’ lifestyles changed positively. - The change in eating behaviors, physical activity and stress management, personal biological and psychological factors, interpersonal and situational influences coincide with the assumptions and propositions of the Health Promotion Model by Nola Pender.	- to measure lifestyle changes and describe the barriers and facilitators perceived that influence on adopting healthy lifestyles in people with cardiovascular diseases.
Sousa, 2021^19^	N = 150 participants	Portugal	Cross sectional study	- FLQ	- ALL	- Self-regulation had direct effects on healthy habits and mental health and indirect effects on well-being and mental health mediated by healthy habits. - Healthy habits exerted direct effects on well-being perception and mental health. - Self-regulation seems to be a good indicator of adopting a healthy lifestyle and better mental health and well-being in the context of the COVID-19 pandemic.	- to explore the mediation role of self-regulation on health-related behaviors adoption or maintenance, mental health, and well-being during the COVID-19 confinement in a sample of adults in Portugal.
Da silva, 2020^20^	N = 103 bus drivers	Brazil	Cross-sectional study	- FLQ	- ALL	- One in four drivers had abdominal obesity and the "Nutrition" and "Type of Behavior" domains were associated with abdominal obesity.	- to estimate the prevalence of abdominal obesity and associated lifestyle factors in bus drivers in a city in Southern Brazil.
Gonçalves, 2020^21^	N = 495 participants	Portugal	Descriptive-correlational study	- FLQ - NQ - BSI	- ALL	- Positive and moderate correlation between nomophobia and psychopathological symptoms. - Interpersonal sensitivity, obsession-compulsion, and the number of hours of smartphone use per day were identified as strong predictors of nomophobia.	- to analyze the propensity of young adults towards nomophobia and lifestyle.
Machul, 2020^22^	N = 444 university students	Poland	Cross-sectional study	- FLQ - SWLS - PSS-10	- ALL	- Polish students obtained higher results in FLQ, and stress levels than foreign students. - The self-assessment of their health condition, lifestyle, and rank associated to being healthy correlated with FLQ, SWLS and PSS-10.	- to analyze the lifestyle practices, satisfaction with life and the level of perceived stress of Polish and foreign students.
Buhrer, 2019^23^	N = 576 university students	Brazil	Cross-sectional, exploratory, descriptive study	- FLQ	- ALL	- Students lifestyle classification was “Good”. However, less than half of the students reported a diagnosis of depression or other chronic mental health condition. - Most of the students did not sleep well and did not feel rested, and not smoked in the last year and had never used drugs. 81% reported an average alcohol intake per week of 0 to 7 units.	- to evaluate the lifestyle and estimate the prevalence of consumption of alcohol, tobacco and other drugs among medical students.
Deluga, 2018^24^	N = 138 elderly people	Poland	Cross-sectional study	- FLQ	- ALL	- The majority of older persons demonstrated an 'excellent' or 'very good' lifestyle with healthy habits.	- to analyze the lifestyle of the elderly in urban and rural areas using the Fantastic Life Inventory.
Lima, 2018^25^	N = 1103 adolescents	Brazil	Cross-sectional and epidemiological study	- FLQ - YRBSQ - COMPAC	- Nutrition - Tobacco	- Efforts to increase levels of physical activity should be focused on older adolescents and those with lower monthly family income.	- to estimate the prevalence of low physical activity levels and to identify related factors in adolescents.
Siu, 2018^26^	N = 118 participants	China	Quasi-experimental	- FLQ - TCUS - DASS-21 - MoCA - DASES	- ALL	- The treatment group showed significant increases in motivation for treatment, reduction in drug use, improvement in cognitive screening tests, healthy lifestyle scores, and self-efficacy in avoidance of drugs over 13 weeks. (However, there were no significant changes in outcome measures covering lifestyle or self-efficacy in drug avoidance.)	- to analyze the outcomes of a short-term hospitalization and support program for people who abuse ketamine.
Bomfim, 2017^27^	N = 672 university students	Brazil	Cross-sectional, observational study	- FLQ - OHQL	- ALL	- Fantastic lifestyle of the Brazilian college students was associated with income, oral health-related quality of life, marital status, religion, and alcohol consumption (binge drinking).	- to analyze possible associations between a Fantastic lifestyle and self-perception of oral health, binge drinking, and socio-demographic variables among public college students.
González-Cantero, 2017^28^	N = 320 university students	Mexico	Cross-sectional and correlational study	- FLQ - GSES - AHS - RQ - LOT	- ALL	- Based on the results of this study, the CapPsi improve lifestyle; however, further research is necessary to determine if the influence of CapPsi is in the adoption and/or maintenance of healthy lifestyle and identify how each one of its factors influences it particularly.	- to determine the relationship between the CapPsi and lifestyle of Mexican university students.
Laguna-Alcaraz, 2017^29^	N = 12 families	Mexico	Transversal study	- FLQ - Clinical, anthropometric and biochemical assessments	- ALL	- The lifestyle was improved after the intervention in the domains of family and friends, nutrition, alcohol intake, and metabolic syndrome.	- to evaluate the impact of a comprehensive intervention, targeting families with teenage sons with overweight and obesity, in the lifestyle, cardiovascular risk factors and metabolic syndrome in a primary care setting.
Martinez-Torres, 2017^30^	N = 890 students	Colombia	Cross-sectional study	- FLQ - Biological measures	- ALL	- The prevalence of MetS was 6.0%, and it was higher in men than women. - The predisposing factors for having a MetS included: being male, over 23 years old, overweight or obese, and having an unhealthy waist-to-height ratio.	- to investigate the prevalence and the associated variables of MetS in Colombian collegiate students.
Oliveira, 2017^31^	N = 143 patients	Brazil	Observational transversal study	- FLQ - Biological measures - VAS	- ALL	- Patients with OA present more sensitivity to pain, more perceived pain, and worse lifestyle than healthy individuals.	- to compare perceived pain, pressure pain threshold, and lifestyle of adult and elderly women with and without knee OA.
Ramírez-Vélez, 2017^32^	N = 1687 university students	Colombia	Cross-sectional study	- FLQ - Biological measures	- Alcohol - Tobacco - Physical activity	- Based on the International Diabetes Federation criteria, both indexes’ thresholds seem to be good tools to identify university students with unfavorable metabolic profiles.	- to explore thresholds of body fat percentage and fat mass index for the prediction of metabolic syndrome among students.
Rodriguez-Gazquez, 2017^33^	N = 140 university students	Spain, Colombia	Cross-sectional descriptive study	- FLQ	- ALL	- The lifestyles are not appropriate in 1 of 3 of nursing students in both universities, - There are statistically significant differences for family items, positive thinkers, the use of safety belts, and alcohol consumption before driving.	- to evaluate the lifestyles of first-year nursing students of 2 universities (one in Spain and the other in Colombia).
Silva, 2017^34^	N = 636 adolescents	Brazil	Cross-sectional epidemiological study	- FLQ - IPAC	- Nutrition	- High prevalence of low health global style HGS levels in adolescents. - Increased HGS levels should be focused on younger boys and normal-weight girls with higher socioeconomic status and lower levels of physical activity.	- to estimate the prevalence of low HGS levels and sociodemographic characteristics, health behaviors and body fatness status related in adolescents.
Tassini, 2017^35^	N = 57 university students	Brazil	Descriptive, cross-sectional, population study	- FLQ	- ALL	- The overall rating was "regular", and none of the participants scored in the "very good" and "excellent” categories. - The domains that mostly required change among medical students related to nutrition and physical activity, while among physical therapy students they related to cigarette, drugs, and alcohol.	- to compare the factors determining the quality of life of students in the healthcare area using the FLQ.
Castro, 2016^36^	N = 930 adolescents	Brazil	Cross-sectional epidemiological study	- FLQ - YRBS - Anthropometric measures	- Sleep	- 1 in 10 adolescents had abdominal obesity; - The associated factors were maternal schooling and television screen time.	- to estimate the prevalence of abdominal obesity and verify the association with sociodemographic factors and lifestyle in adolescents.
Nunes, 2016^37^	N = 916 adolescents	Brazil	Descriptive, cross-sectional and epidemiological study	- FLQ - YRBS	- Nutrition	- Adolescents had a high prevalence of simultaneous risk factors for non-communicable diseases. - Demographic (gender and age) and economic (school shift) variables were associated with the most prevalent simultaneous behaviours among adolescents.	- to investigate the simultaneous presence of risk factors for non-communicable diseases and the association of these risks with demographic and economic factors among these adolescents.
RodríguezGázquez, 2016^6^	N = 380 university students	Colombia	Cross-sectional study	- FLQ	- ALL	- An important proportion of university students has inadequate lifestyles, which means deferred risks for the development of chronic diseases.	- to assess the lifestyles of nursing students from a Colombian public university.
Wilhelm, 2016^38^	N = 366 patients	Australia	Cross-sectional study	- FLQ - SFHS -12 - DASS-21	- ALL	- results support the construct and concurrent validity of the FLQ measure.	- to establish the utility of health-related lifestyle measure (FLQ) in people presenting to a major inner city Emergency Department with a range of suicidal behaviors.
Abdi, 2015^39^	N = 200 employees	Iran	Cross-sectional study	- FLQ	- ALL	- 61.7% of the employees had a favorable lifestyle. - Most of the employees were in a poor condition regarding physical activity and healthy eating habits. - Females got higher scores than males. The associations between lifestyle and age, gender, work experience, income satisfaction, and marital status were significant.	- to evaluate the lifestyle and obesity status of Hamadan public employees and their status based on the trans-theoretical model.
Ramírez-Vélez, 2015^40^	N = 5921 university students	Colombia	Cross-sectional study	- FLQ	- ALL	- “Good lifestyle” was perceived by 57.4% of the females and 58.5% of the males; - In spite of the students being evaluated referring to themselves as having a healthy lifestyle, stated behaviour involving a health risk was observed in the domains concerning nutrition, physical activity and smoking.	- to assess the lifestyle in a sample of university students.
Soares, 2015^41^	N = 1150 adults	Brazil	Cross-sectional study	- FLQ (adapted) - IQ-MSC - WHOQOL	- Stress and ability to relax	- The two approaches to empowerment, the individual and collective are connected, and the physical activity showed to be a good strategy for the empowerment construction.	- to verify the association between individual and collective empowerment with sociodemographic conditions, lifestyle, health conditions and quality of life.
Pacheco, 2014^42^	N = 716 university students	Brazil	Cross-sectional study	- FLQ	- ALL	- Inadequate lifestyle prevalence (5.3 %). - Adjusted analysis results indicated that students over 20 years-old, mothers' formal education had lasted less than nine years, it had a higher risk of having an inadequate lifestyle.	- to determine the association between lifestyle and sociodemographic variables of freshmen attending a state university in southern Brazil.
Silva, 2014^43^	N = 601 adolescents	Brazil	Cross-sectional study	- FLQ - SBCT	- Nutrition - Alcohol	- One-third of the students have low levels of lumbar strength.	- to determine the prevalence and factors associated with low levels of lumbar strength in adolescents.
Silva, 2014^7^	N = 707 university students	Portugal	Cross-sectional study	- FLQ	- ALL	- The instrument demonstrated good overall internal consistency for an instrument used to measure a latent variable. - The FLQ, is a reliable and valid instrument for lifestyle assessment in young adults.	- to make the translation, cultural adaptation and validation of the FLQ in a group of students in higher education in Portugal.
Ferrari, 2013^44^	N = 236 university students	Brazil	Descriptive and cross-sectional study	- FLQ - IPAQ	- Tobacco - Alcohol and drugs - Nutrition	- The prevalence of body image dissatisfaction was 69.5%; 44.1% were dissatisfied with excess weight. - BMI ≥ 25.0 kg/m^2^ was associated with dissatisfaction with excess weight; - Factors associated with dissatisfaction with slimness were being male, eating an unhealthy diet, and smoking tobacco.	- to determine the prevalence of and factors associated with body image dissatisfaction among physical education students enrolled in a public university.
Ramírez-Velez, 2012^45^	N = 550 adults	Colombia	Cross-sectional study	- FLQ	- ALL	- Fantastic questionnaire version 3 brings together some of the internal consistency and construct validity criteria.	- to assess the reliability and validity of the FLQ on a group of Colombian adults.
Silva, 2011^46^	N = 656 adolescents	Brazil	Cross-sectional study	- FLQ	- ALL	- Demographic factors such as school grade, lifestyle habits, low aerobic fitness and excess weight are associated with central obesity.	- to investigate the effects of socioeconomic, demographic and lifestyle factors on abdominal obesity in adolescents from a Brazilian state capital.
Añez, 2008^47^	N = 62 young adults	Brazil	Cross-sectional study	- FLQ	- ALL	- The FLQ has an adequate internal and external consistency for evaluating young adult lifestyles, and it can be recommended for primary care and epidemiological studies.	- to translate and validate the FLQ for use with young adults.
López-Carmona, 2000^48^	N = 103 patients	Mexico	Observational, prospective, longitudinal, and descriptive	- FLQ	- ALL	- The consistency of the instrument is very good. - Their content doesn´t correlate specifically with the control indicators in hypertension.	- to evaluate the reliability and validity of the FLQ, when being applied to a sample of Mexican patients with high blood pressure.
Decina, 1990^49^	N = 81 students	Canada	Cross-sectional study	- FLQ	- ALL	- This preliminary investigation has found differences in lifestyle attitudes in chiropractic students at different levels of training.	- to determine whether healthy lifestyle attitudes are different for students in different years of the chiropractic education process.
Sharratt, 1984^50^	N = 1017 employees	Canada	Cross-sectional study	- FLQ	- ALL	- Employees generally have positive health behaviors. - However, some specific weaknesses were identified such as inactivity, tobacco use among women and older men, and anxiety, worry and depression among women.	- to survey selected health behaviors of University of Waterloo employees.
Simpson, 1984^8^	N = 945 participants	Canada	Cross-sectional and descriptive study	- FLQ	- ALL	- FANTASTIC showed itself to be a reliable lifestyle construct with two major factors: a group of psychosocial behaviors, and a set of "'bad habits".	A survey version of the FANTASTIC Lifestyle Checklist was mailed to a random sample of 1,200 households.

*Note.* FLQ, FANTASTIC Lifestyle Questionnaire; HBP, high blood pressure; ALL, all dimensions; MHD, mental health disorders; TSS, Teaching Stress Scale; BRS, Brief Resilience Scale; OBI, Oldenburg Burnout Inventory; SWLS, Satisfaction With Life Scale; BECCQ, Brazil Economic Classification Criterion Questionnaire; IPAC, International Physical Activity Questionnaire; SBQ, Sedentary Behaviour Questionnaire; HGS, Handgrip strength; FM, fibromyalgia; NQ, Nomophobia Questionnaire; BSI, Brief Symptom Inventory; PSS-10, Perceived Stress Scale; YRBSQ, Youth Risk Behavior Surveillance questionnaire; COMPAC, COMPAC questionnaire; TCUS, Texas Christian University Scales; DASS-21, Depression Anxiety Stress Scale; MoCA, Montreal Cognitive Assessment; DASES, Drug Avoidance Self-Efficacy Scale; OHRQL, oral health-related quality of life; GSES, General Self-efficacy Scale; AHS, Adult Hope Scale; RQ, Resilience Questionnaire; LOT, life orientation test; CapPsi, psychological capital; MetS, metabolic syndrome; OA, osteoarthritis; VAS, visual analogue scale; YRBS, Youth Risk Behavior Survey; SFHS-12, 12-item Short-Form Health Survey; IQ-MSC, Integrated Questionnaire for the Measurement of Social Capital; WHOQOL, World Health Organization Quality of Life-Brief; SBCT, Stages of Behavior Change Tool; BMI, body mass index.

 We analyzed 41 complete articles that fulfilled the inclusion criteria. The studies involved a total of 27 012 participants of different age groups (adolescents, university students, adults, and elderly), and some studies were carried out with families, 495 families were evaluated. The studies analyzed were conducted in different countries such as, Brazil, Colombia, Peru, Spain, Croatia, Portugal, Chile, Poland, China, Mexico, Australia, Iran, and Canada. Several evaluation instruments were used in the studies, but this review aims to analyze a specific instrument – FLQ.

 In this review, the most used dimensions of the instrument were also analyzed, highlighting that most studies use the instrument to assess all dimensions. However, some studies referred to in the literature only use the FLQ to assess specific dimensions such as nutrition,^15,25,37^ sleep,^36^ stress,^41^ tobacco, alcohol and drugs.^32,44^

 A cross-sectional study methodology was clearly evidenced; only two studies referred to a psychometric study of a validation scale.^7,51^

 The literature review showed the continued use of the FANTASTICO instrument until today. This instrument, developed in 1983,^4^ is still used nowadays^10,14^ over different areas of investigation, target groups, objectives and countries. It is a simple, user-friendly instrument that assesses several dimensions. It has proven to be an up-to-date tool, because recently, during this pandemic era, it was used in some studies to diagnose or evaluate the lifestyles of different populations.^11-14,19^ This questionnaire because of its ease of interpretation and quick completion has been used in different populations such as adolescents, youth, college students, adults with different levels of literacy, and the elderly, as shown in Table 1.

## Discussion

 The adoption of healthy lifestyles is key to improving quality of life and preventing disease, and the WHO has been calling attention to this topic over time.^52^ The WHO has stated that a significant percentage of non-communicable diseases could be prevented by people modifying their lifestyles and the associated risk factors, mainly by following a healthy diet and controlling their weight, physical activity, and limiting their alcohol and tobacco consumption.^40,53^

 These results are in line with other investigations, which emphasized that lifestyle is influenced by the habits, attitudes, conduct, traditions, activities, and decisions of a person, or a group of people, regarding the various circumstances in which human beings develop in society or during their daily work/career and which are capable of being modified.^14,40,54^ The domains making up lifestyles would include behavior and preferences related to the type of diet/nutrition, physical activity, drinking alcohol, smoking and/or taking other drugs, responsibility for health, interpersonal relationships, sexual practice, work-related activities/career and consumption patterns.^40,48,54,55^

 Several lifestyle indicators determine people’s health, ranging from social to economic aspects, including environmental and cultural factors, personal attitudes or individual behavior, genetic and physiological characteristics and opportunities.^22,40,42,44^ So, the WHO advocates the incorporation of multilevel such environmental, social, economic, governmental, legal, and interpersonal influences on individuals’ health and behavior.^22,56^

 Although some evidence has shown that adopting and maintaining a healthy lifestyle can improve health, most people have difficulty in adopting a healthy lifestyle.^42,57^ This way, it is necessary to develop interventions especially aimed at the creation or the enhancement of healthy behaviors and toward the reduction of those conducts that are health risks.^57^ The promotion of healthy lifestyles improves people’s quality of life, physical and mental health, well-being and self-actualization.^13^

 In the literature, these results emphasize not only the importance of diagnosing and achieving healthy lifestyles but also underscore the need to implement interventions to promote HL, aimed at increasing self-acceptance and the adoption of healthy behaviors. In this literature review, we have made a retrospective analysis of the use of the FLQ. There is evidence that a questionnaire is an adequate tool for diagnosis and assessing the lifestyles of several populations and should be used in primary care and epidemiological studies to improve people’s quality of life and health.^7^ Nevertheless, much still needs to be done, particularly in increasing literacy about the importance of adopting healthy lifestyles.

## Limitations

 The methodology selected for inclusion and exclusion of these studies always conditions the results obtained, leaving out many valid data studies. We found some limitations in the articles analyzed: the cross-sectional design of the study; the target population should be extended to different age groups; a not representative sample of the population; the difficulty of comparing the results of other investigations. On the other hand, this instrument has limitations, such as not providing accurate assessment since they cover various aspects constituting lifestyle and individuals may not be accurate in their responses, thereby masking reports of inadequate/unsuitable behavior.

## Conclusion

 Lifestyle has been recognized as an important (perhaps the most) determinant of health status (overall in the future of the community) and has become a focus of increasing research interest worldwide.

 This systematic review intended to draw attention to the importance of healthy lifestyles and their diagnosis by use of FLQ, a simple and comprehensiveness, of easy access, and with a quantitative result score for individual ranking, associated with specific counselling. We concluded that this questionnaire is still in use today, in several countries, and among a variety of audiences. It is also possible to verify the relevance of its use because knowing the lifestyles is fundamental to designing intervention strategies for a healthy community. Diagnosis and intervention strategies should be adopted by governments or health entities for a healthy society. Despite the scarce funding for intervention programs for the promotion of healthy lifestyles, it is essential to be alert to this issue and promote HL on this topic.

 In this sense, the use of FLQ could become a pre-diagnosing instrument and a preventative tool to evaluate specific risk behaviors in several populations. Enhancing the diagnosis for subsequent development of intervention strategies in the community.

## Competing Interests

 The authors declare no conflict of interest.

## Ethical Approval

 Not applicable.

## Funding

 The present publication was supported by CEDH, through the CEECINST/00137/2018 and Project UIDB/04872/2020 of the Fundação para a Ciência e a Tecnologia, Portugal. Also, the CIMA was supported by the Fundação para a Ciência e a Tecnologia, project UID/04674/2020.
